# Supplementary figures and images for: Decreasing mitochondrial RNA polymerase activity reverses biased inheritance of hypersuppressive mtDNA
Source: PLoS Genet. 2021 Oct 19;17(10):e1009808. doi: 10.1371/journal.pgen.1009808 (PMC8555793; doi:10.1371/journal.pgen.1009808)

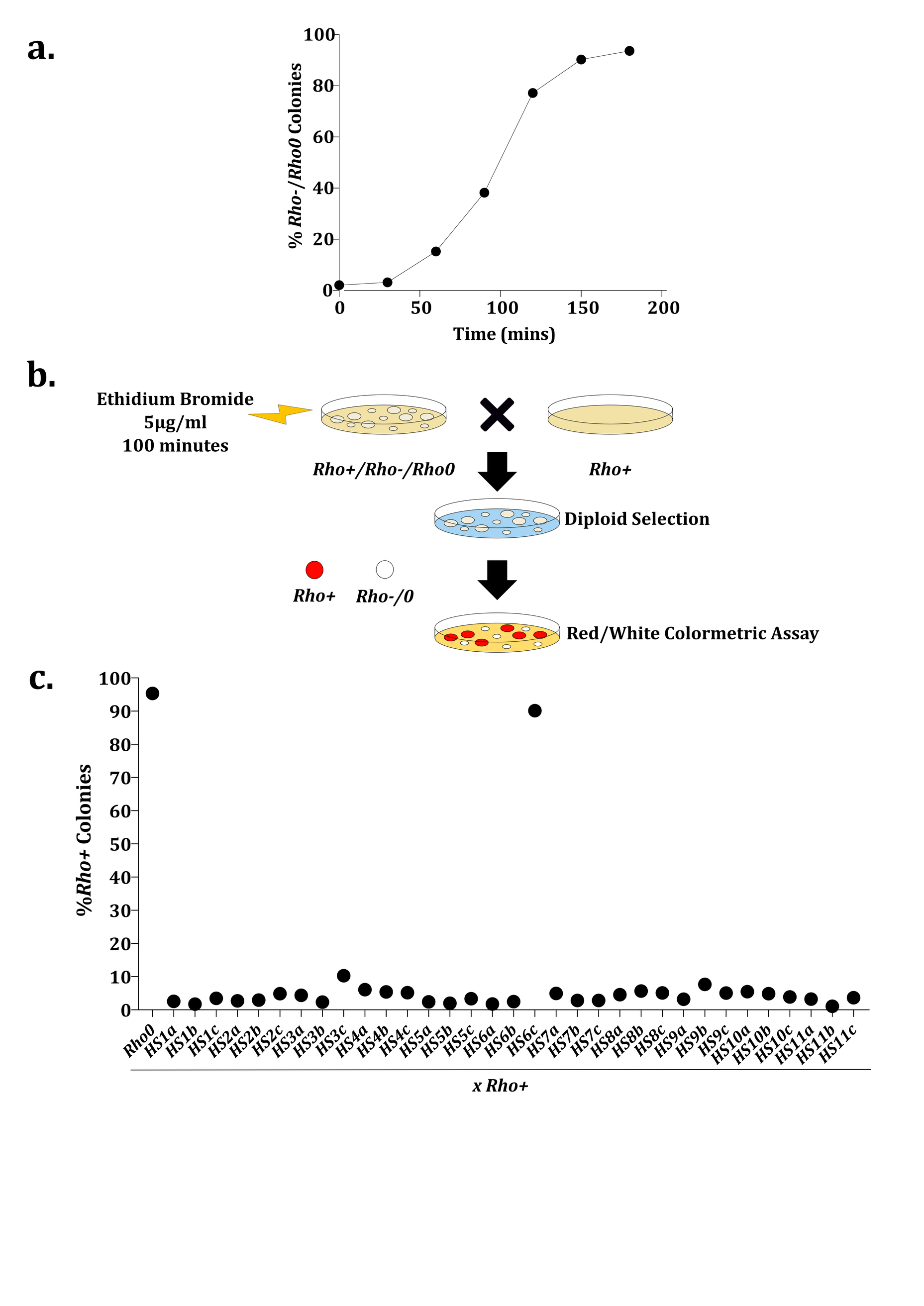

Supplement: S1 Fig — A. 5 μg/ml EtBr was added to rho+ cells and cells were collected every 30 minutes for 180 minutes. Colonies were assessed for respiration. Percent rho- or rho0 colonies was calculated by the formula 100 x [1- (respiratory colonies/total colonies)]. B. Diagram of screen for HS alleles. Rho+ cells were treated with 5μg/ml EtBr for 100 minutes to create a mixed mtDNA population, plated to single colonies on YEPD (1% yeast extract, 2% peptone, 2% glucose), mated with lawns of rho+ yeast on YEPD, selected for diploids on SD-His-Leu, and plated on YEPD plates with no added adenine. Red colonies on the low adenine YEPD plate are rho+ and white colonies are rho- or rho0. White colonies were taken for further analysis. C. HS candidates tested for mtDNA inheritance bias by quantitative mating assay. HS candidates or a rho0 control were mated with a rho+ tester and selected for diploids colonies which were assessed for rho+ mtDNA. (TIF) [file pgen.1009808.s001.tif]

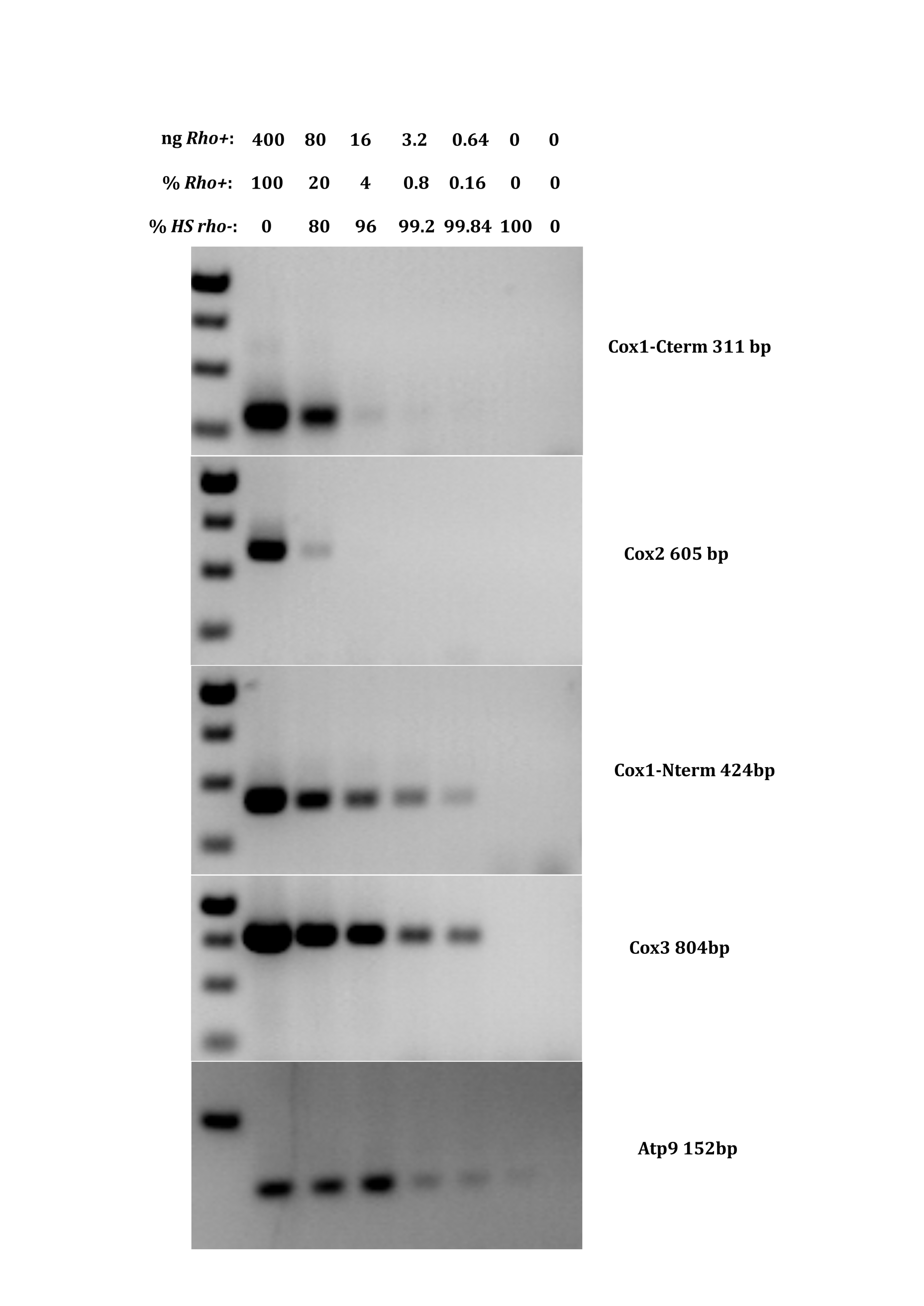

Supplement: S2 Fig — Genomic DNA was isolated from rho+ or HS ORI5-1 parent patches and normalized. Rho+ genomic DNA was diluted into HS ORI5-1 genomic DNA in five-fold serial dilutions. PCRs of using primer sets recognizing mitochondrial loci were performed from the serial dilutions and run on agarose gels containing ethidium bromide. The first lane contains the ladder and the last lane is a no DNA control. (TIF) [file pgen.1009808.s002.tif]

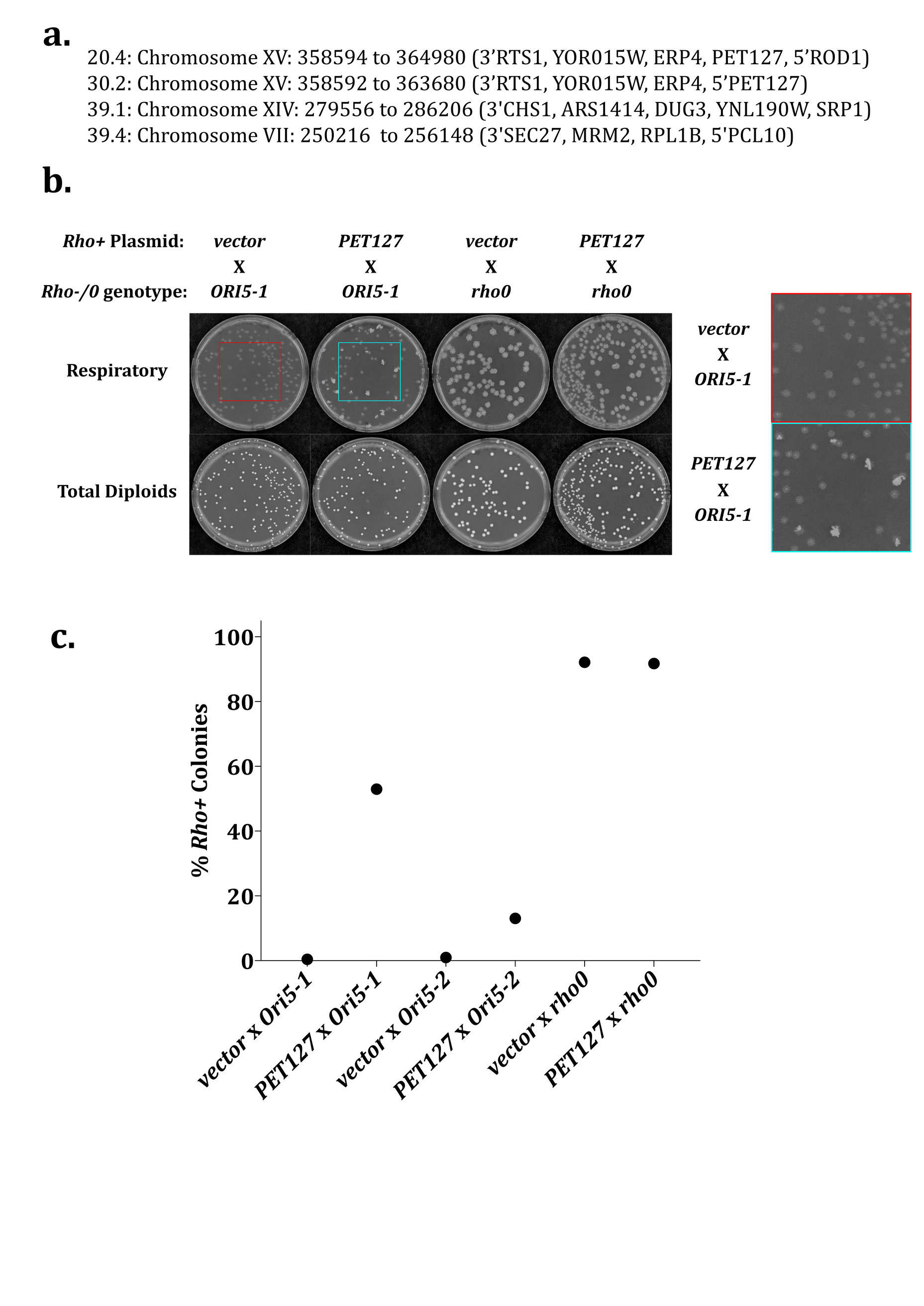

Supplement: S3 Fig — A. Inserts of four plasmids obtained from the high-copy suppressor screen were PCRed using Q5 (New England Biolabs) and Sanger sequenced using the Yep13 backbone primer set. The resulting sequences were identified via NCBI blast [63] and the genomic boundaries of each plasmid insert are as indicated. Parentheses indicate annotated genes contained within the genomic region with 3’ or 5’ indicating that the genic region was cut off and only the 3’ or 5’ end of the gene was present. B. The suppressive capability of the high-copy PET127 plasmid was tested on HS ORI5 alleles cytoduced into a common recipient strain so as to confirm that the extent of high-copy PET127 suppression is determined by the HS allele and not by a possible nuclear mutation. C. Typical plates following quantitative inheritance assay. “Total Diploid” images taken 2 days after plating on SD-His-Leu diploid selection medium. “Respiratory” images taken 3 days after replica plating to YEPG. Inset showing colony section growth. (TIF) [file pgen.1009808.s003.tif]

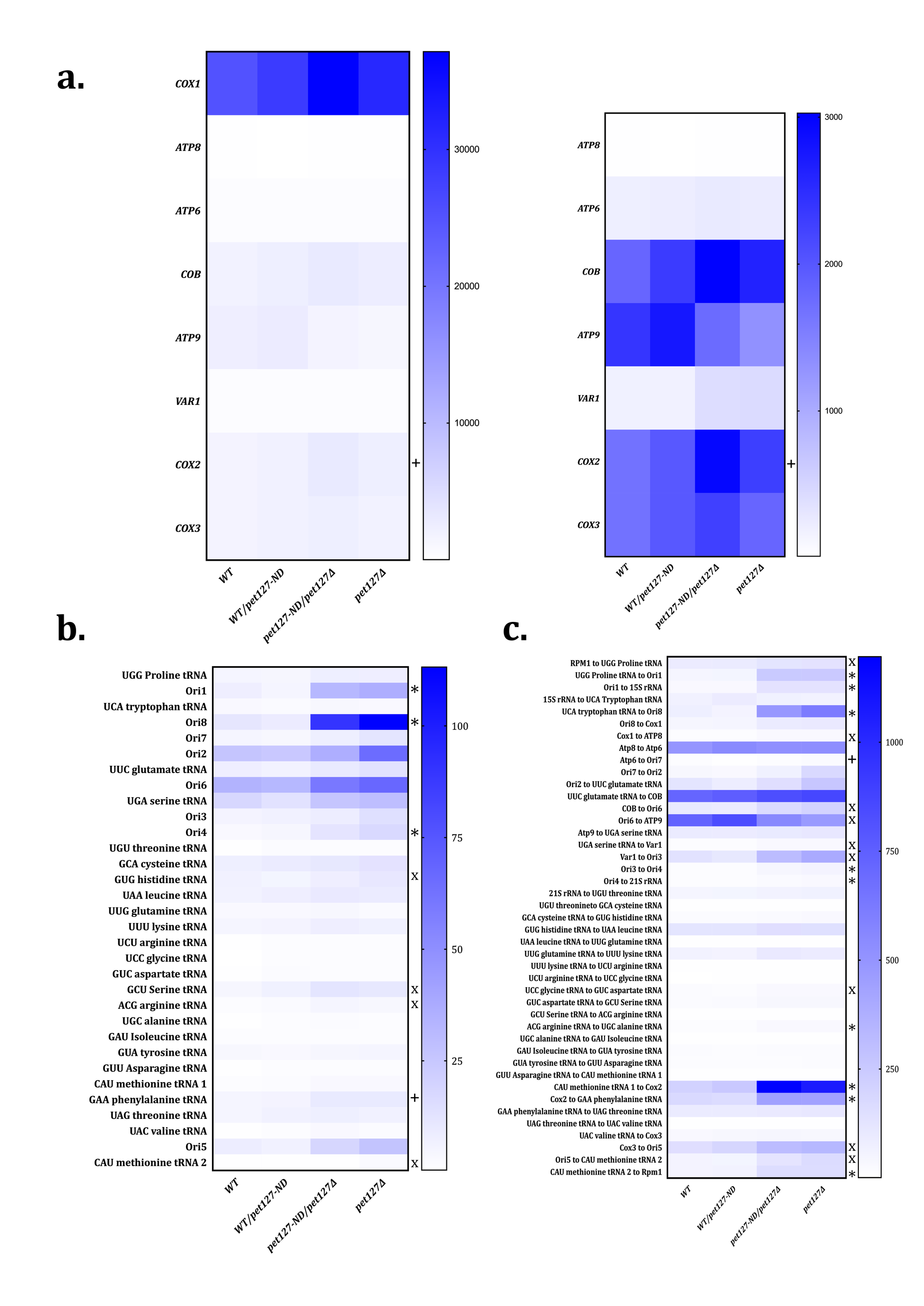

Supplement: S4 Fig — Mitochondrial gene expression analysis of A. Intragenic mtDNA regions with and without COX1 as COX1 has much higher expression relative to the other mitochondrial genes. B. Non-coding RNA regions: tRNA and ORI. C. Intergenic mtDNA regions. * indicates a discovery in both comparisons WT to pet127-ND/pet127Δ and WT to pet127Δ via multiple unpaired T-tests with Two-stage step-up (N = 3). “x” indicates discovery in only WT to pet127Δ. + indicates discovery in only WT to pet127-ND/pet127Δ. No discoveries were identified in WT to WT/pet127-ND. Region delineations can be found in Table 4. (TIF) [file pgen.1009808.s004.tif]

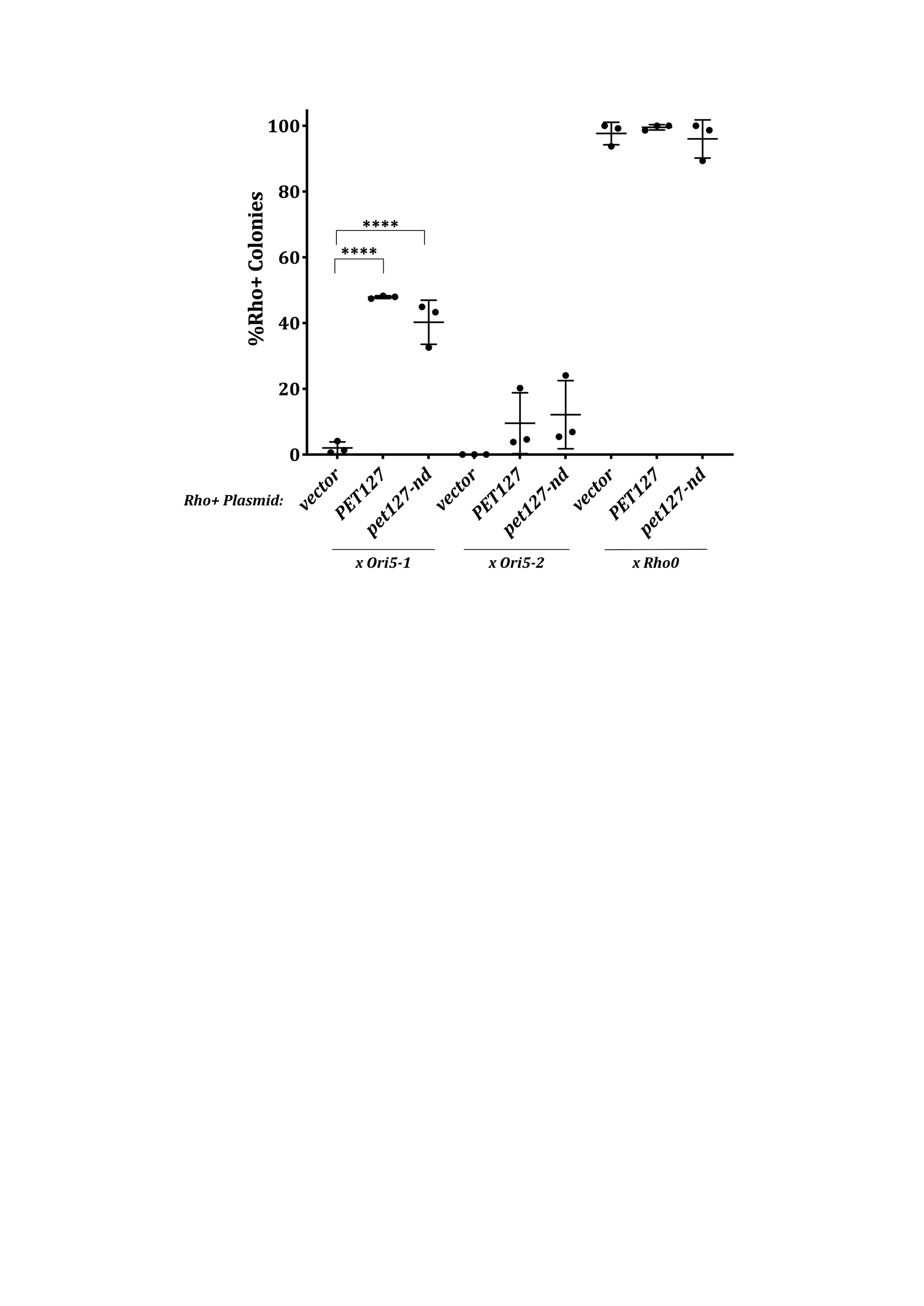

Supplement: S5 Fig — Quantitative mtDNA inheritance assay with high-copy pet127-nd. Significance was determined using one-way ANOVA separately on each HS and rho0 cross with means compared to the vector control using Dunnett’s multiple comparison’s test (N = 3). **** indicates adjusted P-value less than 0.0001. All other comparisons were not significantly different. (TIF) [file pgen.1009808.s005.tif]

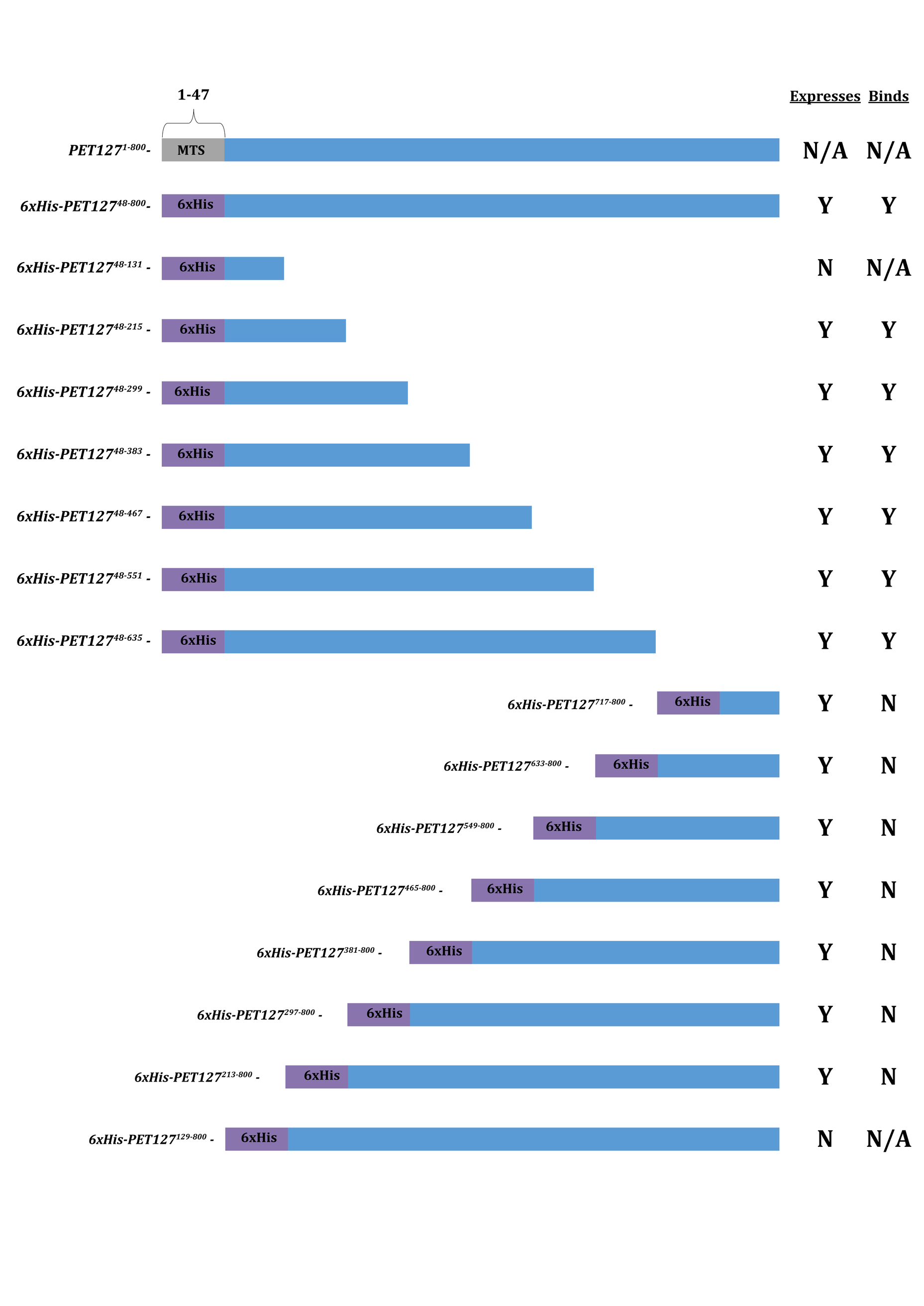

Supplement: S6 Fig — Diagram of tested PET127 truncation constructs in the bacterial expression and binding assay. First row is full length PET127 with predicted mitochondrial targeting sequence. Second row is with the mitochondrial targeting sequence replaced with 6xHis. All the following rows are truncation alleles to scale. Columns indicate whether the allele was able to express in bacteria or subsequently bind with Rpo41 as in Fig 4B. (TIF) [file pgen.1009808.s006.tif]

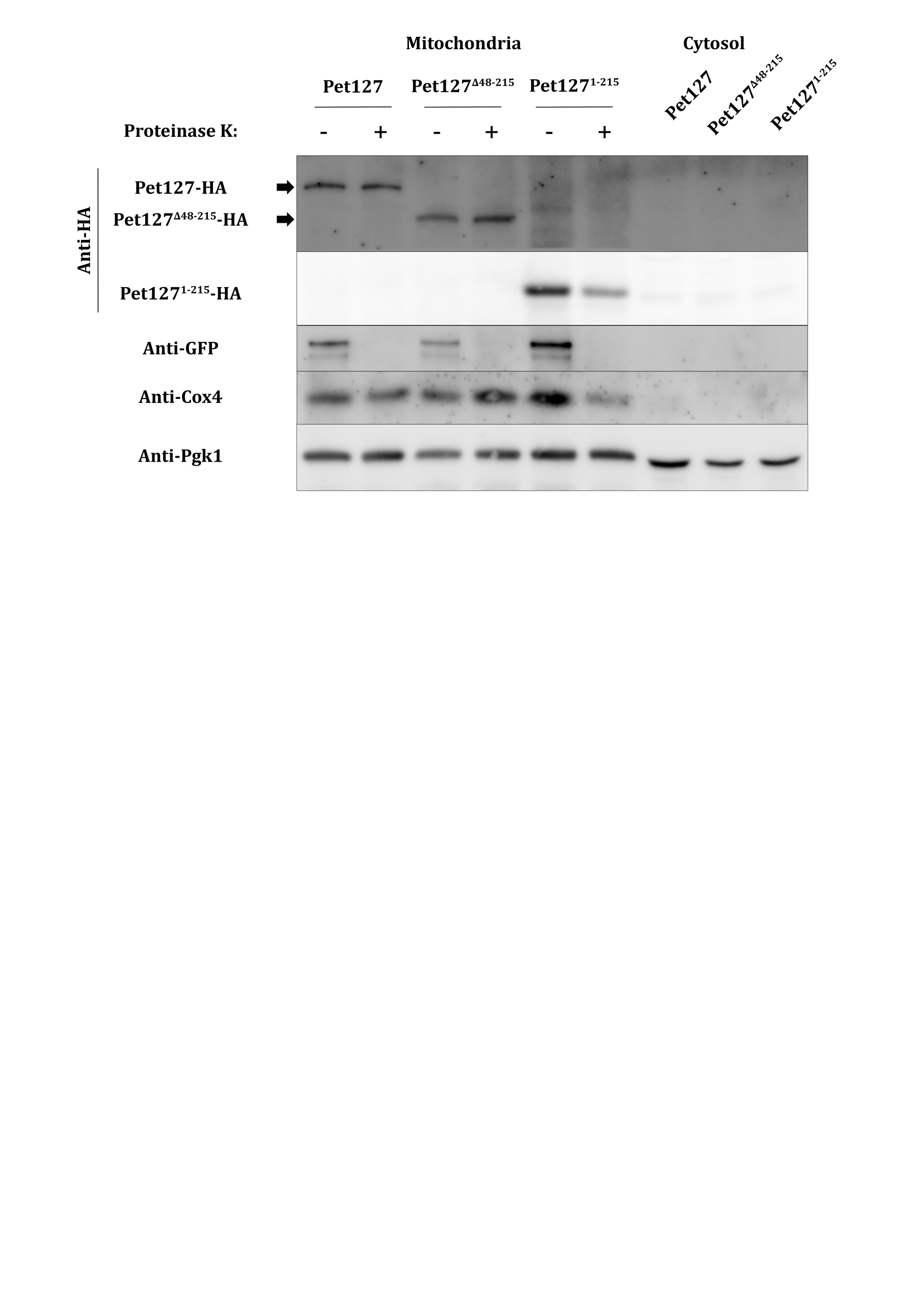

Supplement: S7 Fig — Cycling mutant PET127 and TOM70-GFP carrying cells in YEPD were lysed by zymolyase treatment and Dounce homogenization. Mitochondria was enriched by differential centrifugation and 3μg of mitochondria was treated with 50μg/ml proteinase K for 5mins at 37°C. The reaction was stopped by addition of TCA to 12.5%. Samples were immunoblotted as indicated. A low exposure was used for Pet1271-215-HA because it was more abundant than Pet127-HA and Pet127Δ48-215-HA. Arrows indicate the expected size of Pet127 full length and Pet127Δ48-215-HA respectively. (TIF) [file pgen.1009808.s007.tif]

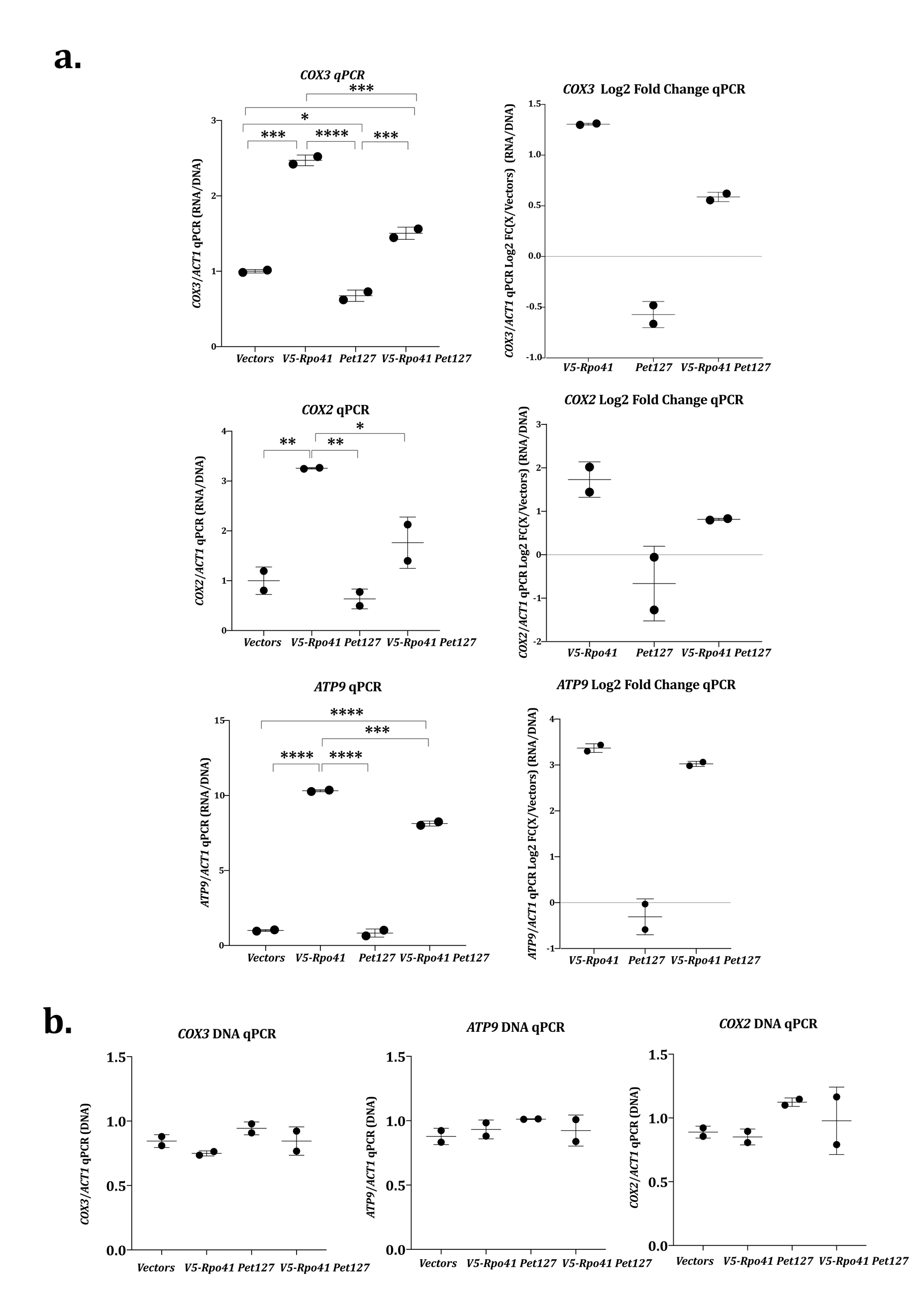

Supplement: S8 Fig — A. RT-qPCR of either COX3, COX2 or ATP9 divided by ACT1 RNA and normalized by the qPCR of the respective gene divided by ACT1 DNA. RT-qPCRs were performed on the high-copy PET127 high-copy RPO41 combination strains collected at high cell density to increase respiration. The left RT-qPCRs are normalized to the Vectors averages. The right column is the same data visualized as a log2 fold change relative to the Vectors control. Significance was determined using one-way ANOVA comparing all values with each other using Tukey’s multiple comparison’s test (N = 2). **** indicates adjusted P-value less than 0.0001, *** indicates an adjusted P-value less than 0.001, ** indicates an adjusted P-value less than 0.01, * indicates an adjusted P-value less than 0.05. B. qPCR of either COX3, COX2, or ATP9 divided by ACT1 qPCR on DNA isolated from high-copy PET127 high-copy RPO41 combination strains. No comparisons were determined as significantly different. Significance was determined using one-way ANOVA comparing all values with each other using Tukey’s multiple comparison’s test (N = 2). (TIF) [file pgen.1009808.s008.tif]

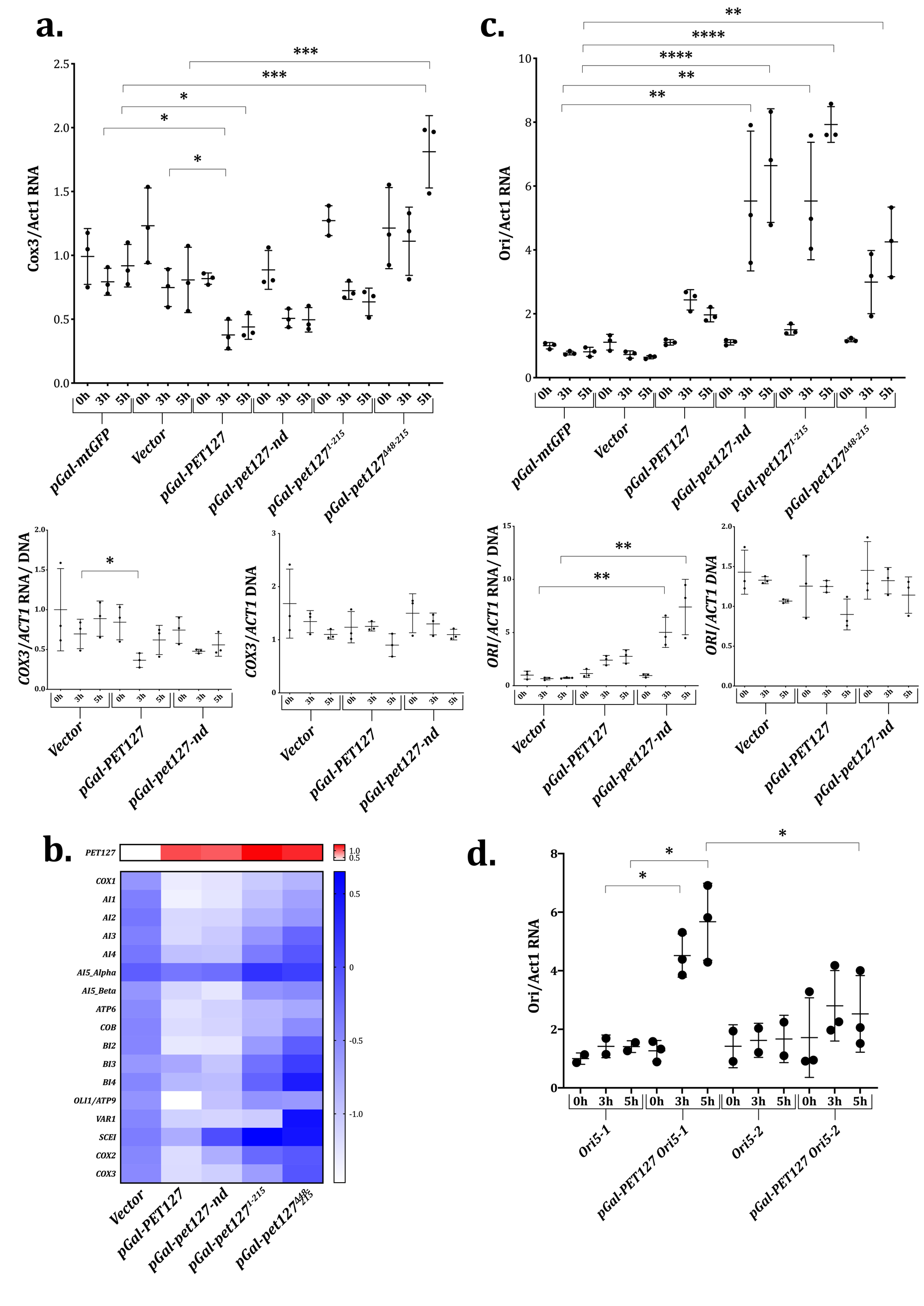

Supplement: S9 Fig — A. COX3 RNA RT-qPCR, COX3 DNA qPCR, or COX3 RNA RT-qPCR divided by COX3 DNA qPCR on high-copy pGal-Pet127 alleles in rho+ cells normalized to ACT1 RNA and/or DNA 0, 3, and 5 hours after addition of galactose. Significance was determined using one-way ANOVA separately for each time point with means compared to either the vector control or the pGal-mtGFP control using Dunnett’s multiple comparison’s test. *** indicates an adjusted P-value between 0.001 and 0.0001. * indicates an adjusted P-value between 0.05 and 0.01. All other comparisons were not significantly different. B. Gene expression analysis via RNA sequencing of PET127 or mitochondrial genic transcripts in cells carrying high-copy pGal-Pet127 alleles. Values indicate average log2 fold change between 0 hours and 5 hours after addition of galactose for three independent experiments. C. ORI RNA RT-qPCR, ORI DNA qPCR, or ORI RNA RT-qPCR divided by ORI DNA qPCR on high-copy pGal-Pet127 alleles in rho+ cells normalized to ACT1 RNA and/or DNA 0, 3, and 5 hours after addition of galactose. Significance was determined using one-way ANOVA separately for each time point with means compared to either the vector control or the pGal-mtGFP control using Dunnett’s multiple comparison’s test (N = 3). **** indicates an adjusted P-value less than 0.0001. ** indicates an adjusted P-value between 0.01 and 0.001. All other comparisons were not significantly different. D. ORI RNA RT-qPCR on HS ORI5-1 and HS ORI5-2 with pGal-Pet127 RNA normalized to ACT1 0, 3, and 5 hours after addition of galactose. Significance was determined using student’s T-test comparing equivalent time points between strains containing either the same ORI or pGal-PET127 allele (N = 2 or 3). * indicates a P-value between 0.05 and 0.01. All other comparisons were not significant. (TIF) [file pgen.1009808.s009.tif]

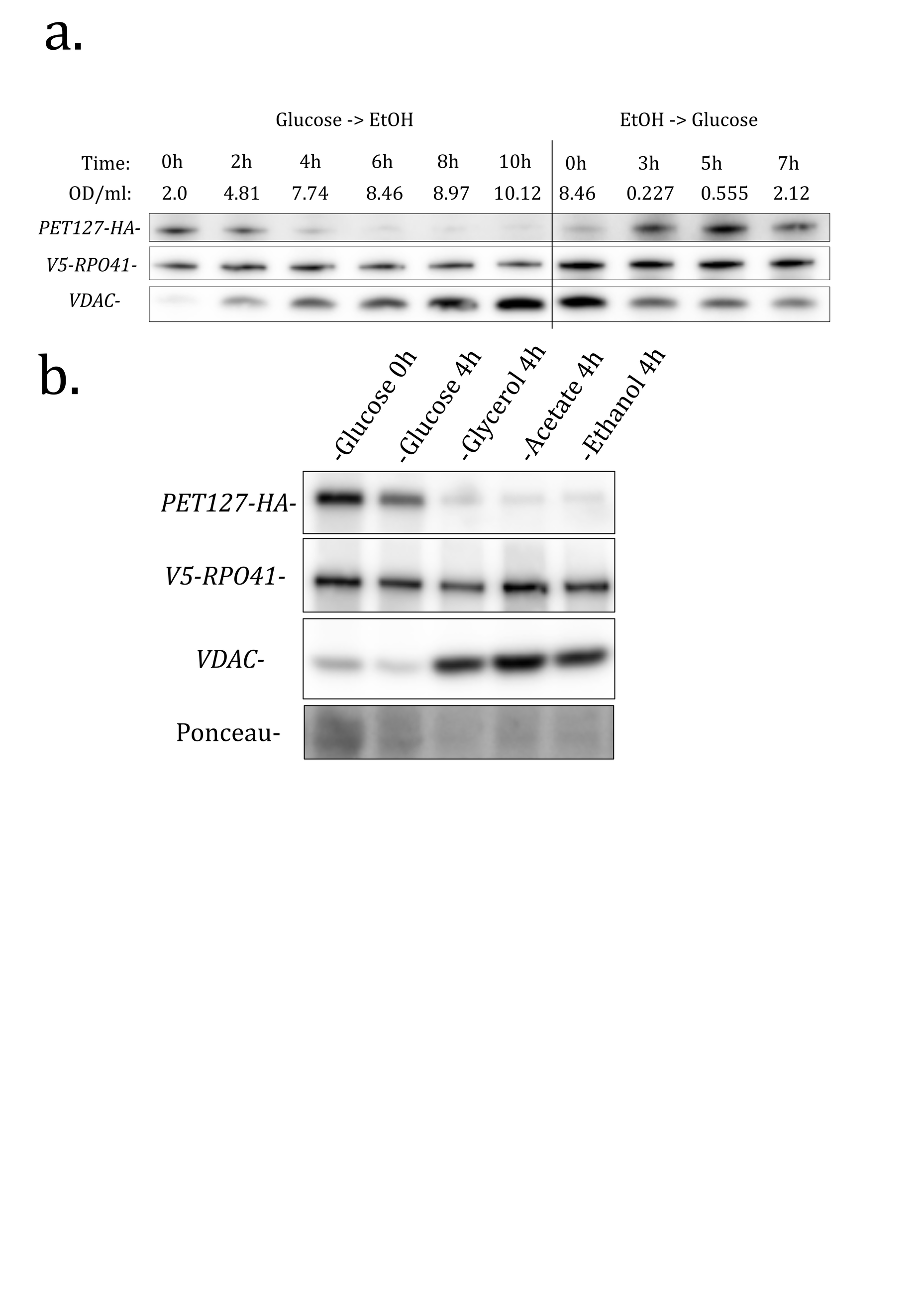

Supplement: S10 Fig — A. Left panel: PET127-HA V5-RPO41 cells were grown to high cellular density in YEPD medium and samples were collected and immunoblotted for HA, V5 and VDAC. Right panel: A high density culture of PET127-HA V5-RPO41 cells in YEPD medium was diluted at 0h to 3.3 x 10^6^ cells /ml in YEPD medium and samples were collected over time and immunoblotted for HA, V5 and VDAC. B. PET127-HA V5-RPO41 cells were grown in YEPD medium to mid log, a sample was taken and then the culture was split into YEP medium containing either 2% Glucose, 2% Glycerol, 2% Potassium Acetate, or 2% Ethanol. Samples were taken after 4 hours and samples were stained with Ponceau S and immunoblotted for HA, V5 and VDAC. (TIF) [file pgen.1009808.s010.tif]

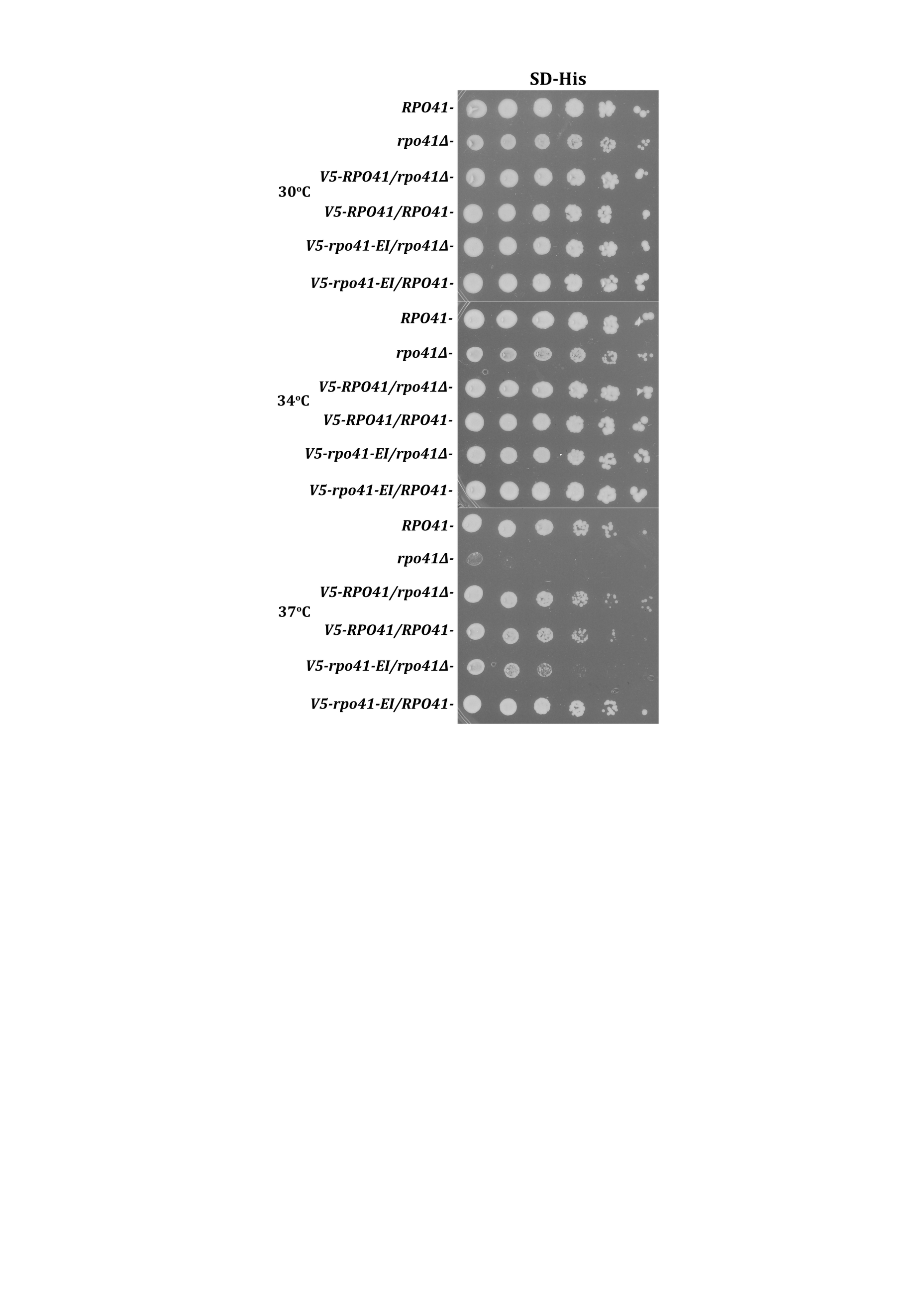

Supplement: S11 Fig — Five-fold serial dilutions of strains containing RPO41 alleles beginning at 1.1 x 10^7^ cells/ml plated on SD-His and incubated at either 30°C, 34°C, or 37°C for 2 days. (TIF) [file pgen.1009808.s011.tif]
