# Supplementary material for: Decreasing mitochondrial RNA polymerase activity reverses biased inheritance of hypersuppressive mtDNA
Source: PLoS Genet. 2021 Oct 19;17(10):e1009808. doi: 10.1371/journal.pgen.1009808 (PMC8555793; doi:10.1371/journal.pgen.1009808)
Supplement: Attachment — Submitted filename: Responses to Reviewer comments Plos Genetics.docx [file pgen.1009808.s012.docx]

Dear Dr Corbi,

Thank you very much for submitting your Research Article entitled 'Decreasing Mitochondrial RNA Polymerase Activity Reverses Biased Inheritance of Hypersuppressive mtDNA' to PLOS Genetics.

The manuscript was fully evaluated at the editorial level and by independent peer reviewers. The reviewers appreciated the attention to an important problem, but raised some substantial concerns about the current manuscript. Based on the reviews, we will not be able to accept this version of the manuscript, but we would be willing to review a much-revised version. We cannot, of course, promise publication at that time.

The reviewers found the identification of pet127 as a modulator of uniparental inheritance for select hypersuprressive petites interesting and were also intrigued by remnants of rho+ DNA in the resulting diploid progeny from the rho+ x HS rho- crosses.    They felt the manuscript could be significantly strengthened with more quantitative analysis describing the loss of rho+ DNA;  controls for mtDNA template abundance when assessing transcription;  and more precise wording so as not to overstate any of the conclusions.

Should you decide to revise the manuscript for further consideration here, your revisions should address the specific points made by each reviewer. We will also require a detailed list of your responses to the review comments and a description of the changes you have made in the manuscript.

If you decide to revise the manuscript for further consideration at PLOS Genetics, please aim to resubmit within the next 60 days, unless it will take extra time to address the concerns of the reviewers, in which case we would appreciate an expected resubmission date by email to [plosgenetics@plos.org](mailto:plosgenetics@plos.org).

If present, accompanying reviewer attachments are included with this email; please notify the journal office if any appear to be missing. They will also be available for download from the link below. You can use this link to log into the system when you are ready to submit a revised version, having first consulted our [Submission Checklist](http://journals.plos.org/plosgenetics/s/submit-now#loc-submission-checklist).

To enhance the reproducibility of your results, we recommend that you deposit your laboratory protocols in [protocols.io](http://protocols.io/), where a protocol can be assigned its own identifier (DOI) such that it can be cited independently in the future. Additionally, PLOS ONE offers an option to publish peer-reviewed clinical study protocols. Read more information on sharing protocols at <https://plos.org/protocols?utm_medium=editorial-email&utm_source=authorletters&utm_campaign=protocols>

Please be aware that our [data availability policy](http://journals.plos.org/plosgenetics/s/data-availability) requires that all numerical data underlying graphs or summary statistics are included with the submission, and you will need to provide this upon resubmission if not already present. In addition, we do not permit the inclusion of phrases such as "data not shown" or "unpublished results" in manuscripts. All points should be backed up by data provided with the submission.

While revising your submission, please upload your figure files to the [Preflight Analysis and Conversion Engine](http://pace.apexcovantage.com/) (PACE) digital diagnostic tool.  PACE helps ensure that figures meet PLOS requirements. To use PACE, you must first register as a user. Then, login and navigate to the UPLOAD tab, where you will find detailed instructions on how to use the tool. If you encounter any issues or have any questions when using PACE, please email us at [figures@plos.org](mailto:figures@plos.org).

PLOS has incorporated [Similarity Check](http://www.crossref.org/crosscheck.html), powered by iThenticate, into its journal-wide submission system in order to screen submitted content for originality before publication. Each PLOS journal undertakes screening on a proportion of submitted articles. You will be contacted if needed following the screening process.

To resubmit, use the link below and 'Revise Submission' in the 'Submissions Needing Revision' folder.


We are sorry that we cannot be more positive about your manuscript at this stage. Please do not hesitate to contact us if you have any concerns or questions.

Yours sincerely,

David M. MacAlpine
Guest Editor
PLOS Genetics

Gregory P. Copenhaver
Editor-in-Chief
PLOS Genetics


Reviewer's Responses to Questions

**Comments to the Authors:
Please note here if the review is uploaded as an attachment.**

Reviewer #1: When mitochondrial genomes undergo a significant deletion event but retain at least one active replication origin, crosses of these haploid cells with ρ+ haploid cells of the opposite mating type will rise <5% ρ+ diploid progeny, a phenotype termed “hypersuppressive”. Such research is fundamental because deleted mtDNA has a replication advantage over wild-type mtDNA in heteroplasmy within a cell.
People usually think that the hypersuppressive mtDNA has a replicative advantage over the rho+ mtDNA. Thus, “Hypersuppressive mtDNA exerts a toxic effect on rho+ mtDNA” is a new concept. For me, it is exciting.
However, after reading this article, I have to say the manuscript still needs improvement by adding more data before consideration for publication.

Major questions:
(1) How can HS mtDNA actively eliminate regions of the rho+ genome?
“regions of rho+ mtDNA persisted after hypersuppressive takeover, indicating that hypersuppressive preferential inheritance may partially be due to the active destruction of rho+ mtDNA.”
We need evidence, not based on speculation. Recombination-deficient mutants reduce HS-biased inheritance by recombinase-driven mtDNA replication. We need more explanation on an imagined recombination DNA destruction mechanism.

We tried a few experiments looking for rho+-HS recombination hybrids and a reduction of those hybrids in Pet127 overexpression strains but couldn’t get consistent results. Establishing a recombination-based destruction mechanism is outside the scope of the paper.

(2) Is Pet127 an inducible protein? When does it inhibit the mitochondrial RNA polymerase RPO41.

Pet127 is inducible. It gets downregulated in respiration conditions and induced in glucose medium. Added figure S10 to show this effect. Likely cells don’t want an inhibitor of RPO41 around during respiration.

Minor questions:
Page 5, line74~line 77:
In most cases, mating between yeast parents containing rho+ mtDNA and parents holding rho- mtDNA results in a moderately suppressive phenotype.
(1) What does entirely respiration competent progeny mean? We should not ignore moderately suppressive phenotype.

Fair, we altered the text to include description of moderate suppressives.

(2) Why can Pet127 protein affect more on wild-type mtDNA but not on HS rho- mtDNA?

Our assumption is that you are asking about the PET127 effect on genic RNA compared to ORI RNA. We primarily looked at the RNA by qPCR so PET127 may affect something qPCR is unable to detect. The data suggest that PET127 affects genic RNA levels more. Possibly RPO41 behaves differently at genic promoters compared to ORI promoters, but why that protects against HS biased inheritance is unclear.

Reviewer #2: PLOS Genetics D-21-00569

The authors of this manuscript have re-examined one of the oldest puzzles in yeast genetics: why to do some deleted versions of mtDNA, termed hypersuppressive (HS) rho- mtDNAs (or petites), eliminate complete rho+ mtDNAs when cells bearing these two forms are mated to produce diploids. Two competing, but not mutually exclusive, models have been considered: the HS mtDNAs might simply out-replicate the rho+ mtDNAs, or the HS mtDNAs might interact with and destroy the rho+ mtDNAs. The replicative interpretation is generally favored, and as a result the sequences found in HS mtDNAs have been termed ORI for origins of replication.

This paper demonstrates that a balance between the interacting proteins Pet127 (a mitochondrial RNA exonuclease) and Rpo41 (the mitochondrial RNA polymerase) is necessary for the full hypersuppressive effect, and that this appears to depend upon normal levels of transcription activity. The authors also present evidence that hypersuppressivity involves destruction of rho+ mtDNA, although this is problematic.

For this study the authors generated a collection of HS strains and showed that, consistent with previous studies, they contain only tandem repeats of three mtDNA sequences known as ORI2, ORI3 and ORI5. The bulk of their experiments were done with those containing ORI5.

The most interesting and novel of the results here flow from their discovery that overexpression of Pet127 reduces the hypersuppressivity of Ori5-1 HS mtDNA. Pet127 is a mitochondrial RNA exonuclease, and is known to physically interact with the mitochondrial RNA polymerase Rpo41 (which the authors confirm here). The authors show that Pet127 nuclease activity is not required for reduction of hypersuppressivity by mutating its active site. Instead it works through binding with the mtRNA polymerase Rpo41. Overexpression of both Pet127 and Rpo41 does not lead to reduction in hypersuppressivity. Thus, there is a balance between these proteins in wild-type: when it is disturbed by Pet127 overproduction hypersuppressivity is reduced. The experiments with truncated forms of Pet127 establish the domain required for this interaction.

The authors state that the effects of Pet127 overproduction are much less evident when tested against ORI2 and ORI3 containing HS strains. The text cites Fig. 3d for evidence, but that figure is missing from the paper (although its legend is there). It appears therefore, that it may not be possible to generalize about the mechanisms of hypersuppressivity.

Major points:
1) There are significant weaknesses in the interpretations of the experiment of Fig. 2a (this should be Fig 2 since there is no 2b). This experiment interrogates multiple diploid clones generated by mating HS-Ori5-1 with rho+ cells, for retention of various sequences mtDNA. These clones comprise populations of cells generated by events occurring during zygote formation and roughly 20 mitotic generations thereafter, and may each contain cells of multiple mitochondrial genotypes. It seems that some of these clones contain rho- mtDNAs that have retained large pieces of rho+ mtDNA. For example Fig. 2a shows that colony 5 lacks Cox2, Cox3 and Cox1-Cterm, but has ATP9, and Cox1-Nterm, while colony 8 retains far more Cox1-Nterm than Cox1-Cterm. (Since they are diploids, it is not possible to test whether these cells retain HS activity.) Thus, it is not surprising that these colonies are respiration incompetent, contrary to page 10, line 173. The authors state (Pg. 10 line 180) "From these data we conclude that rho+ mtDNA is lost in a piecemeal fashion rather than all at once." This implies that they interpret the results as a kinetic analysis. Instead, it is an endpoint analysis of processes that occurred in the zygotes and during the mitotic cell divisions that produced colonies from the zygotes. To generate a time-resolved picture of these events, one would have to follow the fates of mtDNAs during zygote formation and early cell divisions, perhaps using SMRT sequencing of bulk DNA or even single zygotes/cells.

We know the starting DNA profiles in the two parents and the assumption is that the end profile is entirely the HS genome as described in the literature. So, seeing a different DNA profile early on in the process implies seeing an intermediate. This question is very interesting but likely requires a single cell assay to explore which is outside the scope of the paper.

2) The authors employ a temperature sensitive mutation in Rpo41 to test whether its activity affects hypersuppressivity of HS-ORI5. Activity was normal at permissive temperature (30°), but somewhat reduced at an intermediate temperature (34°). The experiment cannot be done at nonpermissive temperature since rho+ mtDNA is unstable in the absence of transcription. The authors conclude that Rpo41 activity is "required" for hypersuppressivity. This is an over-interpretation of the data. The extended discussion of how reduced Rpo41 activity could reduce the hypersuppressiveness of ORI5-containing mtDNAs is overly long and speculative.

Correct that “required” is too definitive of a word. Altered the text to say a reduction of Rpo41 activity reduces hypersuppressiveness of Ori5-1 and reduced the long speculation in discussion.

3) Discussion pg. 22, line 442: The authors write: "The replicative advantage model fails to account for how the rho+ mtDNA vanishes from cells. This is problematic because a heteroplasmic cell having both rho+ and HS mtDNA should be able to respire, as the rho+ mtDNA should behave in a dominant way within a cell." It is not obvious that is true: the heteroplasmic state is extremely unstable in baker's yeast due to the observation of rapid mitotic segregation. This segregation, thought to be due to the seeding of buds with only a few molecules of mtDNA, leads rapidly to pure mitochondrial genotypes from heteroplasmic mother cells. Once the heteroplasmic mother cells become senescent due to replicative aging they will no longer grow on any medium. If the HS rho- mtDNA has a replicative advantage it would be preferentially passed on during mitotic segregation.

FISH data showing equal segregation of DNA hybridizing with rho+ and HS probes into the first bud of a zygote and some rho+ hybridization after 8 divisions (macalpine et. al. 2001) implies that rho+ mtDNA is segregated outside of the zygote.

We have also observed inheritance of genomic regions originating from the rho+ for at least 3 generations via microscopy using a mitochondrial “GFP dots” system developed in Osman et. al. 2015. The dark top side of the zygote is a HS Ori5-2 parent and the bottom is a rho+-lacO parent. Mt-LacI-GFP foci can be detected in the top left cell in the bottom right panel which is three generations removed from the zygote.

If replication is the only factor, the replication disparity would have to be very large to completely disallow segregation of Rho+ genomes. And, if you don’t disallow rho+ genomes then the rapid purification of mitochondrial genotypes will generate pure rho+ progeny which should be present in the population. The only published data on replication rate, as far as we know, is the Chambers and Gingold paper on partial suppressives which didn’t show great disparity in replication rates.

We did some edu incorporation experiments which didn’t show increased replication rate for Ori5-1 or Ori5-2 and no effect of pet127 on the replication rate. This had to be done in a rather contrived system (permanent cell cycle arrest) and there could be a myriad of technical reasons as to why we don’t see the expected effects.

Minor points:

1) Reference 41 (Chambers & Gingold) does not list the year or journal.
An oversight which is now fixed.

2) Fig 2a What is the band amplified by the ATP9 primers that shows up in the rho0 control and is variable in the other lanes? (There is no Fig 2b, so this should just be Fig.2.)

We did not determine the identity of this band as it is larger in size and thus will be amplified less preferentially, and the presence of the band doesn’t change the interpretation of these data. Fixed figures with no “b” throughout the paper.

3) As noted above, Fig. 3d is missing from the manuscript.
It was in the initial paper, the d was maybe too close to the other panel. In this version of the paper, we moved fig3c to the supplemental and expanded the scope of the former figure 3d (now 3c).

4) Fig. 4f: the host yeast strain(s) transformed with overexpressing plasmids in the experiment of Fig. 4f are not indicted in the strain table. Were these strains expressing wild-type PET127? If so, could that have played a role in assisting the truncated Pet127 protein interact with Rpo41?

My apologies, fig 4f used to be fig 4e in a previous version of the paper and I forgot to update the table to reflect this change.

They were expressing wt PET127. It is possible but very unlikely that wild-type PET127 helps the truncation in a non-stoichiometric way as PET127 itself affects biased inheritance in a stoichiometric way. The most likely way non-stoichiometric help would happen is if the exonuclease activity is necessary for PET127 reduction of biased inheritance but not in a stoichiometric way (as we showed stoichiometric exonuclease effects to be uninvolved with the nuclease dead mutant). We find non-stoichiometric exonuclease effects scenario unlikely given that reduction of polymerase activity affects HS and the exonuclease activity seems to promote processing of RNAs for translation which are opposite effects on functional RNA. For these reasons, we would expect see a 2 wt PET127 copy reduction in effect, but did not perform this experiment due to time constraints.

5) Figs 1a and 1b (but not 1c) could be moved to the supplemental materials as they do not represent novel findings or contain information necessary to understand the subsequent text.
Done.

6) Does the absence of Pet127 in diploids have any effect on hypersuppressivity?

A difficult question to answer. we have done this experiment once and it didn’t reduce hypersuppressivity but the expectation genetically is that it might enhance hypersuppressivity. The issue is that hypersuppressivity is already 95% penetrant as a phenotype so enhancement is difficult to assess to statistical significance.

Reviewer #3: The manuscript by Corbi and Amon reports the role of PET127 in the biased segregation of a yeast hypersuppressive (HS) mitochondrial (mt) mutant genome (HS ORI5-1) when mated to the wild type rho+ mitochondrial genome. In an overexpression screen they found that high copy number plasmids containing PET127 increase the fraction of mitotic descendants from the heteroplasmid zygote that retain the rho+ genome. Work from others suggested that Pet127 is a mt exonuclease that is responsible for processing 5’ ends of mt RNAs and that it binds to the mtDNA RNA polymerase (encoded by RPO41). Corbi and Amon confirmed the physical interaction with Rpo41. They made an exonuclease-null mutation of Pet127 and showed that this mutation results in the accumulation of intergenic mtRNA sequences. They physically mapped the region of Pet127 that binds to Rpo41. In crosses with HS ORI5-1, overexpression of the nuclease null mutant but not the Rpo41 binding mutant reduced the inheritance of the HS ORI5-1 genome. They propose that it is Pet127’s inhibition of mtRNA transcription by Rpo41 that is responsible for the improved recovery of rho+ genomes from the heteroplasmic zygotes and support this hypothesis by co-overexpressing both PRO41 and PET127. In addition, they follow the fate of the mt rho+ genome in the descendants of the heteroplasmic zygotes by PCR and find that remnants of the rho+ genome can be detected in the descendants, suggesting that the HS ORI5-1 genome promotes physical destruction of the rho+ genome.

Suppressiveness refers to the propensity of a massively deleted version of the mt genome (a rho- genome that is amplified in head-to tail concatomers) to out-compete the rho+ genome in vegetative growth following mating. The degree of suppressiveness varies on the specific region and/or size of the region of the rho+ genome that is amplified. A few region of the rho+ genome give rise to the hypersuppressive phenotype, but only when the amplicon size is extremely small. These regions contain sequences that have been named rep or ori. It is one specific HS petite that was used to identify Pet127 in this study. The phenomenon of hypersuppressive mitochondrial petites has been around for a long time, but the mechanisms responsible are still unresolved, in part because different HS petites behave differently.

In this manuscript, the authors found an interaction between Rpo41 and Pet127 that influences the HS phenotype of one specific petite (HS ORI5-1) but also show that it clearly doesn’t have the same effect on inheritance of two other HS petites (ORI2-1 and ORI3-1). Moreover, it doesn’t affect HS ORI5-2’s inheritance, even though this larger rho- (with a different junction sequence) contains all of the sequences present in HS ORI5-1. Unfortunately, the authors tend to overgeneralize their findings and the reader is left with the misconception that they have uncovered a universal HS mechanism. I don’t believe it is deliberate, but it would help if the authors were more specific when making conclusions and also talked about some of these puzzling properties of HS petites.

A major concern

The last third of the paper describes transcript abundance data for various mutants and overexpression scenarios. What I find missing from these studies is an estimate of the template abundance. There could be difference is the number of copies per cell of the mtDNA templates that influences the abundance of transcripts but that doesn’t necessarily reflect RNA polymerase activity. The normalizations they perform are comparing ACT1 mRNA to ACT1 DNA and mtRNA to its mtDNA template. But no comparison of mtDNA to ACT1 DNA is made. The copy number of mtDNA can vary and be influenced by state of growth and mutant background. The gal-inducible experiments suffer less from this concern as the cells are only able to go through ~3 doublings (?) so there is less time for a copy number change, but even so, copy number should be checked. I have the same concerns about the ts-RPO41strain grown at the semi-permissive temperature.

In the 2µ overexpression experiments, the qPCR calculation is (mtgene X RNA/ act1 RNA) / (mtgene X DNA/ act1 DNA), so the normalization to mtDNA is baked into that calculation. The DNA levels themselves don’t change all that much and we added a subsection to what was figure S7 with the DNA qPCR data for the 3 mitochondrial loci.

For the Gal-Pet127 experiment, we didn’t have the matching DNA pair for all replicates. We tried repeating the experiment but we believe the raffinose or galactose went bad as we saw no induction of Pet127 upon gal addition. We included the DNA for the replicates we do have and saw no significant DNA loss in the time period assayed. DNA loss will occur if given enough time in galactose as Gal-PET127 24 hr expression causes complete loss of mtDNA. Wiesenberger and Fox showed this with 2µ pADC1-PET127 plasmids causing loss of mtDNA even after reintroduction of Rho+ mtDNA.

The concern about the mtDNA levels in rpo41-ts at the semi-permissive temperature is misplaced. We only switch to the semi-permissive temperature after mating is complete and cells are plated onto the diploid selection medium. So, any differences in DNA at the permissive temperature prior to mating will also be present in the permissive temperature controls, and any differences observed in the semi-permissive temperature after mating are real effects on biased inheritance. There could be some concern that we’re seeing loss of HS mtDNA instead of effects on biased inheritance, but it is still affecting HS mtDNA more than *rho+* whereas the current thinking is that *rpo41∆* is thought to allow for stable HS mtDNA and unstable *rho+*.

Other concerns:

1) The authors frequently draw attention to changes in measurements that don’t reach significance. That puzzles me and I find it misleading.

We removed all observations related to data which did not reach significance.

2) The authors need to be more precise when summarizing their results as their findings specifically affect only one HS petite genome and not the other three they isolated.

We included data (fig 3c) showing PET127 suppression of other genome’s biased inheritance. We limited the scope of conclusions where relevant.

3) Describing the HS regions of the rho+ genome as replication origins is an overstatement. People have proposed that they are origins but I do not know of any study that shows that replication actually initiates at these sites. Rolling circle replication started by invasion of a DNA end is a model with considerable support.

We debated whether to outright call them origins when initially writing the paper but some of the literature calls them origins, so we decided to call them origins as well. As they are not necessary for replication, we have removed the origin designation.

4) The finding that regions of the rho+ genome persists is interesting. The only evidence is from PCR which is notoriously non-quantitative. Because so little attention was paid to the specifics of the PCR, I am wondering whether adequate controls were performed to be sure that differences found between primer pairs wasn’t just due to the fact that different primer pairs were used. Did the authors spike in different amounts of the rho+ parent into the DNA from the haploid rho- to convince themselves (and the readers) that the absence of product was biologically real? If this observation is real, it would be worth pursuing by Southern blotting or PacBio sequencing to look for the novel junction sequences.

We performed the spike experiment (figure S2). There were differences between the primers which may have contributed to some but not all of the missing bands.

The Pac bio experiment is tricky as we purified mitochondria to obtain enriched mtDNA and the volume of cells needed for mitochondrial purification is quite a lot. We can’t just grow up the colonies as they will lose the intermediates.

Responses to other concerns:

1. Motivation: What is the rationale for expecting an overexpression screen to affect hypersuppressivity? Is it just a matter of convenience or was there some hypothesis you were testing?

The rationale is that a recessive loss of function screen would be very difficult to perform. It is easier to look for dominant suppressors, so we chose to try an overexpression screen.

2. Conundrum: Why would a gene (PET127) that produces petites when deleted be expected to also influence a petite phenotype when overexpressed? This point bothered me the whole time I was reading through your manuscript.

PET127 doesn’t produce petites when deleted. It wasn’t generated from a petite phenotype screen and was named “pet” for unknown reasons. The initial paper which named the gene didn’t show the overexpression petite phenotype.

3. Reciprocality: The crosses were always done (I think) with the overexpressing plasmid in the rho+ cell. What happens when the reciprocal experiment is performed? Does all of the action happen in the zygote or does the overexpression have some effect on the rho+ genome before mating? (I’m wondering the effect of the plasmids on rho+ copy number that influences the outcome of the mating. For example, does reducing transcription in the rho+ genome drive up copy number pre mating so that there are more competing rho+ genomes in the zygote? That is just one possibility that comes to mind.)

We tried one experiment with 2µ PET127 in the HS background and observed the same results as with PET127 in the rho+ background. We kept PET127 in the rho+ background to dissuade the notion that PET127 causes loss of parental mtDNA prior to mating as prior literature shows that extremely high expression of PET127 causes rho0 formation.

4. Dominance: It isn’t clear whether the wild type allele for PET127 is present in the strains with the plasmid borne version of PET127 when the strain is a parent in one of the crosses. Maybe these are present in the huge strain lists, but I did not dig through these tables to answer my questions. And if the WT allele is present, what does it imply for the conclusions?

We kept wild-type PET127 in the background of all HS crosses. It likely adds two copies of PET127 to the overexpression effects. There is a small possibility that the wild-type PET127 has a non-stoichiometric effect, but this is unlikely as the enzymatic activity of PET127 seems to be promoting RNA processing which is the opposite effect of reduction of polymerase activity.

5. Destruction of the rho+ genome: If your model for HS replacement of the rho+ genome is a result of recombination between the HS genome and the rho+ genome, then there should be Pacbio sequences that show these novel junctions between the tandem repeats of the HS genome and rho+ DNA. Alternatively, wouldn’t carefully planned restriction digests and probing of Southern blots reveal junction fragments to support your claim?

We tried looking for rho+-HS junctions by PCR but had inconsistent results. It’s difficult to probe for Pacbio sequences as we purified mitochondria to enrich for mtDNA and the volume of cells required for that is quite high. Southern restriction digests of colony genomic DNA might work. We didn’t perform this experiment due to time constraints and that establishing a recombination-based mechanism of destruction is outside the scope of the paper.
